# Computational screening for novel *α*-glucosidase inhibitory peptides from *Chlamys nobilis* adductor muscle as a potential antidiabetic agent

**DOI:** 10.3389/fnut.2025.1566107

**Published:** 2025-03-24

**Authors:** Haisheng Lin, Wen Wang, Lei Du, Jialong Gao, Wenhong Cao, Huina Zheng, Zhongqin Chen, Xiaoming Qin, Yuanwei Liang

**Affiliations:** ^1^College of Food Science and Technology, National Research and Development Branch Center for Shellfish Processing, Guangdong Provincial Key Laboratory of Aquatic Products Processing and Safety, Guangdong Ocean University, Zhanjiang, China; ^2^State Key Laboratory of Bioreactor Engineering, Department of Food Science and Engineering, East China University of Science and Technology, Shanghai, China; ^3^College of Chemistry and Environment, Guangdong Ocean University, Zhanjiang, China

**Keywords:** *Chlamys nobilis*, α-Glucosidase inhibitory peptides, molecular docking, glucose tolerance, antioxidant activity

## Abstract

**Introduction:**

This study aimed to evaluate the adjuvant hypoglycemic function of enzyme hydrolyzate (EHCA) from *Chlamys nobilis* in mice and to identify *α*-glucosidase inhibitory peptides.

**Methods:**

The *α*-glucosidase inhibitory and radical scavenging ability of EHCA were determined *in vitro*, and the effects on blood glucose regulation and the antioxidant activity were evaluated *in vivo* using a mouse model. Peptides with potential *α*-glucosidase inhibitory activity were identified by LC-MS/MS and confirmed *in silico*.

**Results and Discussion:**

EHCA exhibited significant *α*-glucosidase inhibitory activity and radical scavenging activity against 2,2-diphenyl-1-picrylhydrazyl (DPPH). *in vivo*, EHCA significantly improved the glucose tolerance of mice, reduced malondialdehyde and increased the superoxide dismutase activity in liver. Five novel peptides were identified, with Lys-Leu-Asn-Ser-Thr-Thr-Glu-Lys-Leu-Glu-Glu and Thr-Asp-Ala-Asp-His-Lys-Phe showing strong inhibitory effects on *α*-glucosidase (IC_50_ value of 144.89 μM and 136.96 μM, respectively). The interactions between peptides and *α*-glucosidase were driven by hydrogen bonds, van der Waals forces, and hydrophobic interactions. These findings suggest that EHCA and its derived peptides could serve as potential adjuvant agents for blood glucose regulation and antioxidant activity. The identified peptides may pave the way for the development of alternative *α*-glucosidase inhibitors.

## 1 Introduction

Diabetes mellitus type 2 (T2DM) is a metabolic disease with impaired insulin secretion and insulin action in the pancreas. Poor diet and lifestyle are the main causes of this serious global health problem (1). Existing conventional drugs to control blood glucose levels such as acarbose, metformin hydrochloride and miglitol have side effects such as susceptibility to drug resistance, causing liver and kidney damage (2). There is growing interest in the use of natural products in pharmacology and nutrition with the aim of alleviating the common side effects associated with the use of synthetic drugs. Previous studies have demonstrated that the inhibition of *α*-glucosidase effectively delays the breakdown of carbohydrates into absorbable monosaccharides, thereby reducing postprandial glucose levels and alleviating hyperglycemia (3, 4). Therefore, *α*-glucosidase inhibitors (AGIs), such as acarbose, voglibose, and miglitol, are commonly used as antihyperglycemic agents (5). Additionally, oxidative stress has been identified as a key factor in the pathogenesis of T2DM, and compounds with antioxidant properties have been shown to mitigate diabetes-related complications by scavenging reactive oxygen species (ROS) and reducing cellular damage. These findings highlight the potential of *α*-glucosidase inhibitors and antioxidants as therapeutic agents in the management of diabetes.

In general, peptides derived from dietary sources have been demonstrated to possess potential bioactivities that have beneficial effects on human health, and their favorable pharmacokinetic properties, including good solubility, low immunogenicity, and low toxicity, allow them to be easily incorporated as functional foods (6, 7). In addition, bioactive peptides have the potential to inhibit target enzymes such as ACE, DPP-IV, and *α*-glucosidase, which may have the effect of preventing and managing the onset of metabolic syndrome by altering metabolic pathways or directly modifying them with target enzymes (8–10).

Marine proteins have a novel amino acid composition, which is one of the important sources for the production of bioactive peptides (11, 12). There have been a number of studies on natural hypoglycemic active peptides from marine sources such as *Trachurus trachurus*, *Euphausia superba*, *Chlorella vulgaris* and *Crassostrea gigas* (11, 13–15). The anti-diabetic peptide USW extracted from *Spirulina* was able to improve insulin resistance by significantly increasing activity of glucose metabolizing enzyme. The peptide LRSELAAWSR obtained from the *Spirulina platensis* showed the high inhibitory activity against *α*-glucosidase (IC_50_ = 134.2 μg/mL) (16). The protein hydrolyzate of *Oncorhynchus kern* skin significantly reduces fasting blood glucose levels and protects pancreatic *β*-cells from apoptosis (17). Overall, AGIP reduces the absorption of glucose from the gastrointestinal tract and improves postprandial blood glucose levels by preventing the cleavage of oligosaccharides and disaccharides into absorbable monosaccharides (18).

Many studies have shown that enzymatically digested peptides from *Chlamys nobilis* have been shown to have antioxidant, anti-cancer (19), and fertility-promoting properties (20). Previous studies found that the enzymatic hydrolyzate from the adductor muscle of the *Chlamys nobilis* possesses *α*-glucosidase inhibitory activity (21), suggesting its potential as a potent novel natural hypoglycemic active substance. However, the potential effects of peptides in enzymatic hydrolyzate on the inhibition of *α*-glucosidase and its adjuvant hypoglycemic function in mice have not been fully investigated.

In this study, we determined the *in vitro* α-glucosidase inhibitory activity and DPPH radical scavenging ability of enzyme hydrolyzate (EHCA) from the adductor muscle of *Chlamys nobilis* by *in vitro* assays and evaluated its adjuvant hypoglycemic function in mice by *in vivo* experiments. Novel peptides with potential *α*-glucosidase inhibition and antioxidant activities were rapidly identified from EHCA by UHPLC-ESI-LC–MS/MS combined with virtual screening, and their activities were further verified by synthesized peptides, resulting in two novel bioactive peptides. This study demonstrated that EHCA has antioxidant activity and potential auxiliary hypoglycemic activity *in vivo* and is a good source of marine nutritional source for the preparation of marine nutritional food.

## 2 Materials and methods

### 2.1 Materials and chemicals

Enzymatic hydrolyzate of *Chlamys nobilis* adductor muscle (EHCA) was obtained from the laboratory following the method mentioned by Lin et al. (21). *α*-glucosidase (200 U/mg) and p-Nitrophenyl-α-D-glucopyranoside (pNPG) were from Shanghai Yuanye Biotechnology Co., Ltd. (Shanghai, China). 1,1-Diphenyl-2-picrylhydrazyl (DPPH) free radical scavenging capacity kit, activity determination kit of superoxide dismutase (SOD)-WST-8 method, Malondialdehydes (MDA) content kit were procured from Suzhou Grace Biotechnology Co., Ltd. (Suzhou, China). Accu-Chek Active Roche blood glucose test paper was sourced from Roche Diagnostic Care GmbH (Shanghai, China). Enzyme-linked immunosorbent assay (ELISA) kit for mouse insulin (INS) were ordered from Jiangsu Yutong Biotechnology Co., Ltd. (Jiangsu, China). Male Kunming mice [body mass (25 ± 3) g] were bought from Zhuhai Bestest Biotechnology Co., Ltd. (Zhuhai, China). All other chemicals and reagents were analytically pure.

### 2.2 Analysis of the *α*-glucosidase inhibitory activity

The inhibition of EHCA or peptides against α-glucosidase was assayed following the methods described elsewhere with some modifications (21). Refer to Table 1 for reagent addition levels, 110 μL of PBS (pH 6.8, 0.2 M), 20 μL of sample, and 20 μL of *α*-glucosidase (3.3 U/mL) were added to each well, respectively. Next, the mixture was placed at 37°C for 10 min, then 20 μL of pNPG (1.25 mmol/L) was added, and the reaction was carried out at 37°C for 20 min. Finally, 80 μL of Na_2_CO_3_ solution (0.1 mol/L) was added to terminate the reaction, and the absorbance at 405 nm was then determined. Three parallel wells were set up in each group.

**Table 1:** α-glucosidase activity inhibition system.

Reagent	Blank/μL	Blank control/μL	Sample/μL	Background control/μL
PBS	110	110	110	110
Sample	–	–	20	20
α-glucosidase	20	–	20	–
Distilled water	20	40	–	20
pNPG	20	20	20	20
Na_2_CO_3_ solution	80	80	80	80

The rate of *α*-glucosidase inhibition (*W_α_*, %) was calculated using the following formula (Equation 1):


(1) $$W_{\alpha }=\left[1-\left(A_{3}-A_{4}\right)\right]/\left(A_{1}-A_{2}\right)\times 100$$

Where *A*_1_ is the absorbance of the blank well; *A*_2_ is the absorbance value of the blank control wells; *A*_3_ is the absorption of the reaction hole of the sample; *A*_4_ is the absorbance value of the sample background hole.

### 2.3 DPPH free radical scavenging ability

The standard curve of DPPH free radical scavenging rate y = 2.8486x + 0.7084 was obtained by diluting FBS with methanol into the following concentration gradients: 0, 5, 10, 15, 20, 25 μg/mL, according to the procedure in the instructions manual of the DPPH free radical scavenging kit. The OD value of the sample at 517 nm wavelength was determined and its DPPH free radical scavenging ability (*Y*, μgTrolox/mL) was calculated by the following equation (Equation 2):


(2) $$Y=0.351\times \left[\left(1-A_{2}+A_{1}\right)/A_{0}–0.7084\right]\times D$$

Where *A*_0_, *A*_1_, *A*_2_ are the absorbance of blank well, blank control well and sample reaction well respectively; D is the dilution ratio of the sample.

### 2.4 Effect of EHCA on blood glucose in normal mice

Fifty healthy adult male Kunming mice were purchased from Zhuhai Bestest Biotechnology Co., Ltd. The experiments were approved by the Animal Ethics Committee of Guangdong Ocean University with the approval number of GDOU-LAE-2021-002, approved on 6 April 2021. Before the experiment, all animals were watered and fed freely for 7 days.

The animals were randomly divided into 5 groups of 10 animals each based on blood glucose values, including the administration group (500, 1,000 and 2,000 mg/kg of body weight, therein called EHCA-L, EHCA-M and EHCA-H, in that order), the positive control group (metformin 150 mg/kg, PC) and the negative control group (distilled water, NC). The groups were mixed with soluble starch (250 mg/kg) and gavaged synchronously for 3 d. After 12 h of fasting, tail vein glucose concentrations were measured using a Roche Vitality Glucometer at 0, 0.5 and 2 h in each group after administration of gavage starch (22).

Fasting blood glucose was measured in mice before and after gavage using Roche Vitality Blood Glucose Test Strips after 12 h of fasting, and the rate of glycemic reduction (*W*_G_, %) was calculated according to the following equation (Equation 3):


(3) $$W_{G}=\left(G_{0}-G_{1}\right)/G_{0}\times 100$$

Where *G*_0_ is the pre-dose fasting blood glucose value, mmol/L; *G*_1_ is the fasting blood glucose at the end of dosing, mmol/L.

Area under the curve (AUC) was calculated applying the following formula (Equation 4):


(4) $$Q_{AUC}=\left(a+4b+3c\right)/4.$$

Where *a*, *b*, and *c* are blood glucose values at 0, 0.5 and 2 h of gavage, respectively, mmol/L.

### 2.5 Effect of EHCA on serum insulin (INS)

Effects of EHCA on mice at the end of the test, blood was taken from the eyeballs of mice, which were executed in the decapitated position, and the organs were collected for subsequent experiments. Obtained fresh blood at room temperature natural coagulation 10–15 min, centrifuged at 10,000 r/min for 10 min, the supernatant was collected, and the absorbance was measured at 450 nm according to the kit instruction manual to draw the standard curve of the quasi-product. The serum insulin (INS) levels in each group of mice were obtained from the curves and the insulin sensitivity index (*W*_ISI_) and insulin secretion index (*W*_FBCI_) were calculated for each group by the following equation (Equations 5 and 6 respectively).


(5) $$W_{ISI}=\ln\left[1/\left(W_{FPG}\times W_{\mathrm{FINS}}\right)\right]$$

(6) $$W_{\mathrm{FBCI}}=W_{\mathrm{FINS}}/W_{FPG}$$

Where *W*_ISI_ is the insulin sensitivity index; *W*_FPG_ is fasting blood glucose, mmol/L; *W*_FINS_ is fasting insulin content (μU/mL); and *W*_FBCI_ is the insulin secretion index.

### 2.6 Effect of EHCA on hepatic malondialdehyde (MDA) and superoxide dismutase (SOD)

Tissue homogenate was prepared by homogenizing 0.5 g of liver with 9 times the mass of saline, and the supernatant was centrifuged at 10,000 r/min for 10 min to determine the MDA content and SOD activity of mouse liver tissue according to the instructions of the MDA and the SOD-WST-8 assay kit.

### 2.7 Characterization of peptide sequences

Mini ultrafiltration module (P2PLBCC01, Millipore, United States; NMWCO 3 kDa) was used to collect the peptide fraction (<3 kDa) in the EHCA supernatant. Then peptide fraction was desalted and analyzed by Nano LC–MS/MS system (EASY-nLC 1,200, Thermo Scientific, Waltham, MA, United States) in conjunction with Q Exactive™ Hybrid Quadrupole-Orbitrap™ Mass Spectrometer (Thermo Fisher Scientific, Waltham, MA, United States). Chromatographic separations were performed on an analytical column (Acclaim PepMap RPLC C18, 150 μm × 150 mm, 1.9 μm, 100 Å, Thermo Scientific, Waltham, MA, United States). Mobile phase A, 0.1% formic acid; mobile phase B, 0.1% formic acid in 80% acetonrtrile; flow rate, 600 nL/min. The peptide sequences were identified using PEAKS Studio 8.5. The parameters were set as follows: the maximum missed cleavages were set to 2; the precursor ion mass tolerance was set to 20 ppm, and MS/MS tolerance was 0.02 Da.

### 2.8 Prediction of physicochemical properties of peptides

Toxic prediction tool ToxinPred was used to evaluate the toxicity of peptides (https://webs.iiitd.edu.in/raghava/toxinpred/index.html, accessed on 18 October 2023). Next, the peptide property calculator (https://www.novopro.cn/tools/calc_peptide_property.html, accessed on 18 October 2023) was used to predict water-soluble peptides. For the follow-up study, the peptides that were easily water-soluble (Gravy value below −1) and non-toxic were selected.

### 2.9 Molecular docking

Peptides were constructed and energy minimized by Chem3D 21.0.0 and then imported into AutoDock Tools 1.5.7 software to add atomic charges, assign atom types, and make all flexible bonds rotatable by default. The X-ray crystal structure of *α*-glucosidase was retrieved from the Protein Data Bank (https://www.rcsb.org, PDB ID: 2QMJ accessed on 16 October 2023) and processed to remove irrelevant small molecules, remove water molecules, add hydrogen atoms, and set the atom type. 2QMJ had active centers of X: −20.83, Y: −6.71, and Z: −5.25 (23). Semiflexible docking was performed by AutoDock Vina, molecular docking results were analyzed, and peptide-protein binding interactions were visualized using Pymol 2.2.0 software.

### 2.10 Molecular dynamics simulations

The molecular dynamics simulation was conducted using GROMACS version 2022.3 (24). For the preprocessing of small molecules, AmberTools22 was utilized to implement the Generalized Amber Force Field (GAFF), while Gaussian 16 W was employed for the hydrogenation of small molecules and the calculation of the Restrained Electrostatic Potential (RESP). The potential data generated will be incorporated into the topology file of the molecular dynamics system. Upon completion of the simulation, the built-in analysis tool of the software was employed to evaluate the trajectory. The analysis included the calculation of the root mean square deviation (RMSD), root mean square fluctuation (RMSF), solvent accessible surface area (SASA), radius of gyration (Rg), and hydrogen bonds for each amino acid trajectory.

### 2.11 Synthesis of *α*-glucosidase inhibitory peptides

Based on the results of the LC–MS/MS analysis and virtual screening, two peptides with purity above 98% (w/w): Lys-Leu-Asn-Ser-Thr-Thr-Glu-Lys-Leu-Glu-Glu (KLNSTTEKLEE), Thr-Asp-Ala-Asp-His-Lys-Phe (TDADHKF) were prepared using the solid phase synthesis method with support from Qiangyao Biotechnology Co., Ltd. The purity of the synthesized peptides was characterized using RP-HPLC coupled to a Kromasil C18 column (100-5C18, 4.6 mm × 250 mm, 5 micron, column temperature, 30°C). RP-HPLC mobile phase A: acetonitrile with 0.1% trifluoroacetic acid, mobile phase B: pure water with 0.1% trifluoroacetic acid at a flow rate of 1 mL/min. The elution gradient was 25% A initially, 50% A within 20 min, and 100% A within 20–20.1 min, and the run was stopped at 25 min with UV detection at 220 nm. Eventually, the purified peptides were identified by ESI-MS spectroscopy.

### 2.12 Statistical analysis

The results were expressed as mean ± standard deviation (SD), One-way analysis of variance (ANOVA) and Duncan’s multiple extreme variance with significance test was performed using SPSS Statistics 22 software, and there was a significant difference at *p* < 0.05. The Graphical Abstract were drawn by Figdraw 2.0.

## 3 Results and discussion

### 3.1 *α*-Glucosidase inhibitory and DPPH radical scavenging effects of EHCA

α-Glucosidase inhibitors could slow the breakdown of starch into glucose by inhibiting the action of alpha-glucosidase in the intestinal mucosa, thereby achieving the effect of reducing and delaying glucose absorption in the small intestine. Therefore, the *α*-glucosidase inhibitory activity could be used as an important indicator for evaluating the hypoglycemic activity (25).

**Figure 1:**
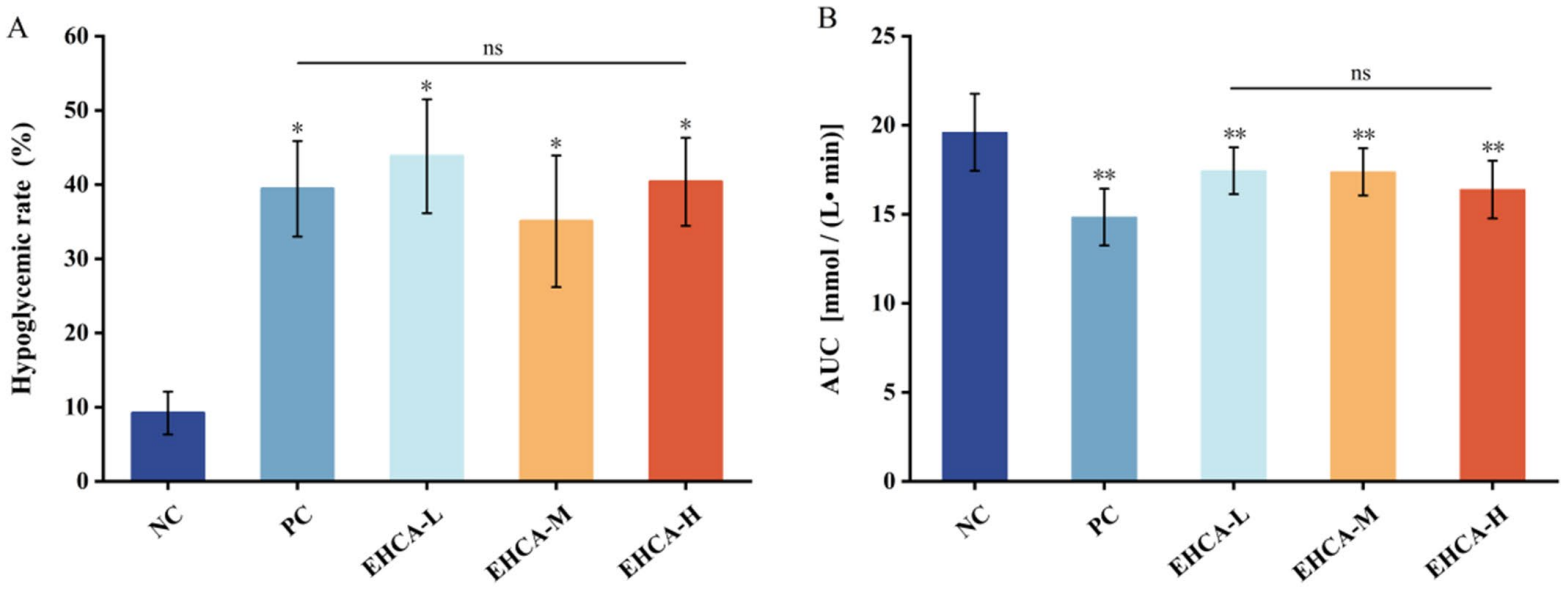
Effect of different concentrations of EHCA on hypoglycemic rate **(A)** and AUC **(B)** of blood glucose in mice. Compared with the negative control group (NC), ^*^p < 0.05, ^**^p < 0.01, “ns” indicates no significant differences among groups at the *p* < 0.05 level, *n* = 10 mice per group. Reproduced from Lin et al. (21), licensed under CC BY-NC-ND 4.0.

The *α*-glucosidase inhibitory activity and DPPH radical scavenging rate of EHCA were detected at concentrations of 15, 30, and 60 mg/mL (Figure 1). As shown in Figure 1A, EHCA displayed excellent DPPH radical scavenging activity in a dose-dependent manner (*p* < 0.05). Furthermore, EHCA inhibited α-glucosidase regardless of peptide concentrations (Figure 1B). Within the concentration range tested, inhibition of *α*-glucosidase ranged from 34.36 to 35.45%.

DPPH radical scavenging activity assay has been widely used in evaluating the antioxidant properties of peptide extracts (26). In our study, the DPPH radical scavenging rate was up to 80.02% with a scavenging capacity of 27.84 μgTrolox/mL under the condition of EHCA concentration of 60 mg/mL. Many previous studies have shown that natural products with antioxidant activity might be equally effective *α*-glucosidase inhibitors (GIA) (26–28). Oxidative stress is an important factor in the development of T2DM. Oxidative stress caused by high ROS levels has been shown to increase intestinal glucose uptake by affecting glucose transporters (SGLT1 and GLUT2), thereby exacerbating diabetes (26, 29).

Therefore, we explored the α-glucosidase inhibitory peptides of EHCA (Figure 1B). The results suggest that peptides derived from the adductor muscle of Chlamys nobilis may reduce oxidative stress and provide potential hypoglycemic health benefits. Below, the effects of EHCA on blood glucose regulation in normal mice and its antioxidant activity *in vivo* were evaluated.

### 3.2 Analysis of *in vivo* adjuvant hypoglycemic activity of EHCA

#### 3.2.1 Effect of EHCA on blood glucose concentration and glucose tolerance in mice

The results of the blood glucose values of the experimental mice given EHCA gavage for 3 d intervention are shown in Table 2, and the fasting blood glucose values (*G*_0_) of the experimental group of mice were all reduced compared to the NC group, but there was no significant difference (*p >* 0.05). After 0.5 h and 2 h of gavage, both the EHCA-delivered group and the PC group were able to significantly reduce the blood glucose values of the mice compared with the NC group (*p <* 0.05), and all of them were the lowest in the PC group, which was manifested by the blood glucose values of (8.56 ± 1.19) mmol/L at 0.5 h and (6.87 ± 1.14) mmol/L at 2 h. These was followed by the EHCA-H group, with blood glucose values of (9.48 ± 1.28) mmol/L at 0.5 h and (7.46 ± 1.43) mmol/L at 2 h. These results indicated that EHCA could enhance the glucose tolerance of mice significantly. As shown in Figure 1A, both the EHCA and metformin significantly increased the hypoglycemic rate in fasted mice compared with the NC group (*p <* 0.05), indicating that EHCA intervention has a significant hypoglycemic effect. However, there was no significant difference in the hypoglycemic effect between different doses of EHCA (*p >* 0.05) (Figure 1A). In addition, relative to the AUC value [(19.60 ± 2.16) mmol / (L • min)] in the NC group of mice, both EHCA and metformin markedly reduced the AUC values (*p <* 0.05) (Figure 1B). It was found that continuous administration of EHCA for 3 d improved the glucose tolerance effect in normal mice, which is consistent with the results reaped in the test of the effect of marine aquatic proteolytic enzymes on glucose tolerance in rats (30).

**Table 2:** Effect of EHCA on blood glucose values in mice.

Group	Blood glucose value / (mmol/L)			
	*G*_0_	0.5 h	2 h	*G*_1_
NC group	4.18 ± 1.33^a^	11.40 ± 1.41^Aa^	9.54 ± 1.14^Aa^	3.70 ± 0.99^a^
PC group	4.94 ± 1.71^a^	8.45 ± 0.91^Cc^	6.87 ± 0.66^Cd^	3.21 ± 0.20^a^
EHCA−L group	4.92 ± 1.27^a^	9.92 ± 0.73^Bb^	8.40 ± 0.90^Bb^	3.26 ± 0.67^a^
EHCA−M group	4.68 ± 1.43^a^	10.10 ± 0.98^Bb^	8.15 ± 0.96^Bbc^	3.26 ± 0.55^a^
EHCA−H group	5.26 ± 1.69^a^	9.48 ± 1.01^BbC^	7.46 ± 0.81^BCcd^	3.45 ± 0.83^a^

a–d superscripts indicate significant differences (*p* < 0.05) and A–C superscripts indicate a highly significant differences in each column (*p* < 0.05), *n* = 10 mice per group.

#### 3.2.2 Effect of EHCA on serum insulin in mice

Table 3 shows the results of normal mouse serum insulin affected by EHCA, with no significant difference between the fasting serum insulin of the groups of mice. Compared with the NC group, the insulin sensitivity index was elevated in each administration group, but there was no significant difference between the groups (*p >* 0.05). The results indicate that EHCA-assisted hypoglycemia is not related to insulin secretion.

**Table 3:** Effects of EHCA on insulin in normal mice.

Group	Fasting serum insulin/(mIU/L)	Insulin sensitivity index	Insulin secretion index
NC group	29.14 ± 4.96^a^	2.14 ± 0.47^a^	9.36 ± 4.36^a^
PC group	29.82 ± 5.50^a^	2.13 ± 0.13^a^	8.50 ± 1.12^a^
EHCA−L group	26.48 ± 6.86^a^	2.16 ± 0.28^a^	8.96 ± 2.32^a^
EHCA−M group	28.34 ± 7.49^a^	2.23 ± 0.30^a^	9.65 ± 2.75^a^
EHCA−H group	29.36 ± 4.62^a^	2.16 ± 0.28^a^	8.97 ± 2.78^a^

Different letters superscripts indicate significant differences in each column (*p* < 0.05), only the letter “a” is used as a superscript, indicating no significant differences among the groups, *n* = 10 mice per group.

#### 3.2.3 Analysis of EHCA on MDA content and SOD activity in mouse liver

**Figure 2:**
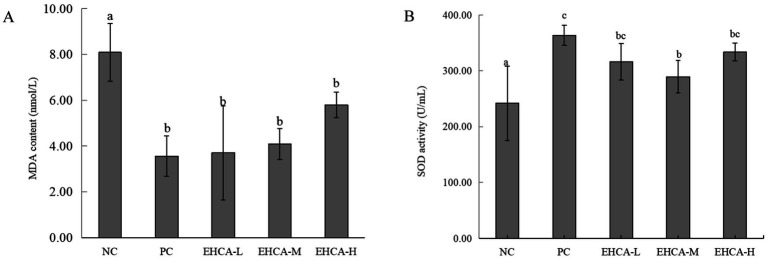
Effect of EHCA on MDA content **(A)** and SOD activity **(B)** in mouse liver. Compared with the negative control group (NC), the means with different letters within the same group have significant differences at the 0.05 probability level, *n* = 10 mice per group. Adapted with permission from Lin et al. (21).

Previous research has shown that the MDA content of diabetes model mice was significantly elevated, and the reduction in MDA content was an effective indicator of hypoglycemic activity (31). The body can play a role in preventing diabetes by scavenging free radicals and thus reducing cell damage and apoptosis (32). The MDA content and SOD activity in mouse liver was detected and the results are listed in Figure 2. The metformin group clearly had the most notable MDA content (*p <* 0.01), followed by the EHCA-L and EHCA-M groups (*p <* 0.05) (Figure 2A). This result is consistent with the findings of Ou (31). The SOD activity in the liver of mice was significantly increased (*p* < 0.01) in all dosing groups and PC group compared to the NC group [(242.00 ± 66.43) U/mL] (Figure 2B). Among them, the SOD activity [(363.55 ± 18.09) U/mL] in PC group was improved by 50.23%. The SOD activity in EHCA-L group [(316.24 ± 65.96) U/mL], EHCA-M group [(289.56 ± 29.29) U/mL], and EHCA-H group [(333.58 ± 65.33) U/mL] were increased by 30.68, 19.63, and 37.84%, respectively, and there was no significant difference between the administered groups (*p >* 0.05). It indicated that EHCA had an enhancing effect on SOD activity at different doses without concentration dependence. Both results show that EHCA possessed significant antioxidant activity *in vivo*.

### 3.3 Identification of peptide sequences

Next, in order to determine which components of EHCA are responsible for the *α*-glucosidase inhibitory activity, LC–MS/MS analysis was performed. Peptide sequence resolution of the raw mass spectrometry files was performed using PEAKS Studio 8.5 software, and a total of 627 peptides were identified in EHCA. The molecular weight of protein hydrolysates is a determinant of their biological activity, and peptide fractions with low molecular weights are highly biologically active (33). The α-glucosidase inhibitory peptides TAELLPR, CGKKFVR, AVPANLVDLNVPALLK and VVDLVFFAAAK, isolated and characterized from the black tea proteins named HMJ and QL, had molecular weight distributions from 798.4 to 1647.8 Da (34). Walnut-derived peptide LPLLR (molecular with 610.79 Da) inhibited *α*-glucosidase by 50.12% (2 mM), suggesting a possible antidiabetic effect of LPLLR *in vitro* (35). Therefore, peptides with score > 56 and lengths <20 amino acids were searched through the website https://webs.iiitd.edu.in/raghava/toxinpred/index.html and https://www.novopro.cn/tools/calc_peptide_property.html for toxicity and water solubility prediction. Five nontoxic peptides with good water solubility (Table 4) were screened, and they were derived from the tropomyosin protein (Protein Accession: Q9GZ69) (Table 4), which is a major protein component in the adductor muscle of *Chlamys nobilis*. Tropomyosin is known for its excellent amino acid composition, which provides a rich source of bioactive peptides upon enzymatic hydrolysis. Previous studies have demonstrated that the enzymatic hydrolysate from the adductor muscle of *Chlamys nobilis* possesses *α*-glucosidase inhibitory activity (21). This finding suggests that the specific amino acid sequence of Tropomyosin enable the formation of peptide fragments with potent antidiabetic potential peptides. And these five potential α-glucosidase inhibitory peptides were found to be unreported (http://www.uwm.edu.pl/, accessed on 18 October 2023), which could be used to further investigate the molecular mechanism of peptide inhibition of *α*-glucosidase (36).

**Table 4:** Identification and docking free energy scoring of α-glucosidase-inhibiting active peptides *in vitro*.

Peptides sequence	RT (min)	*m / z*	Mass	Score	Toxicity	Gravy value	Protein number	Affinity (kcal/mol)
EQKLKDTETAKAK	5.96	745.4135	1488.8147	77.92	Non-toxic	−1.82	Q9GZ69	−6.0
KLNSTTEKLEE	11.81	646.3407	1290.6666	71.33	Non-toxic	−1.49	Q9GZ69	−7.2
VATDADHKFDEAARK	10.08	558.6133	1672.8169	65.78	Non-toxic	−1.07	Q9GZ69	−6.8
TDADHKF	7.97	417.1935	832.3715	58.81	Non-toxic	−1.46	Q9GZ69	−7.9
ISDDLDQTF	33.79	527.2424	1052.4662	56.49	Non-toxic	−0.49	Q9GZ69	−5.9

### 3.4 Virtual screening and molecular docking

**Figure 3:**
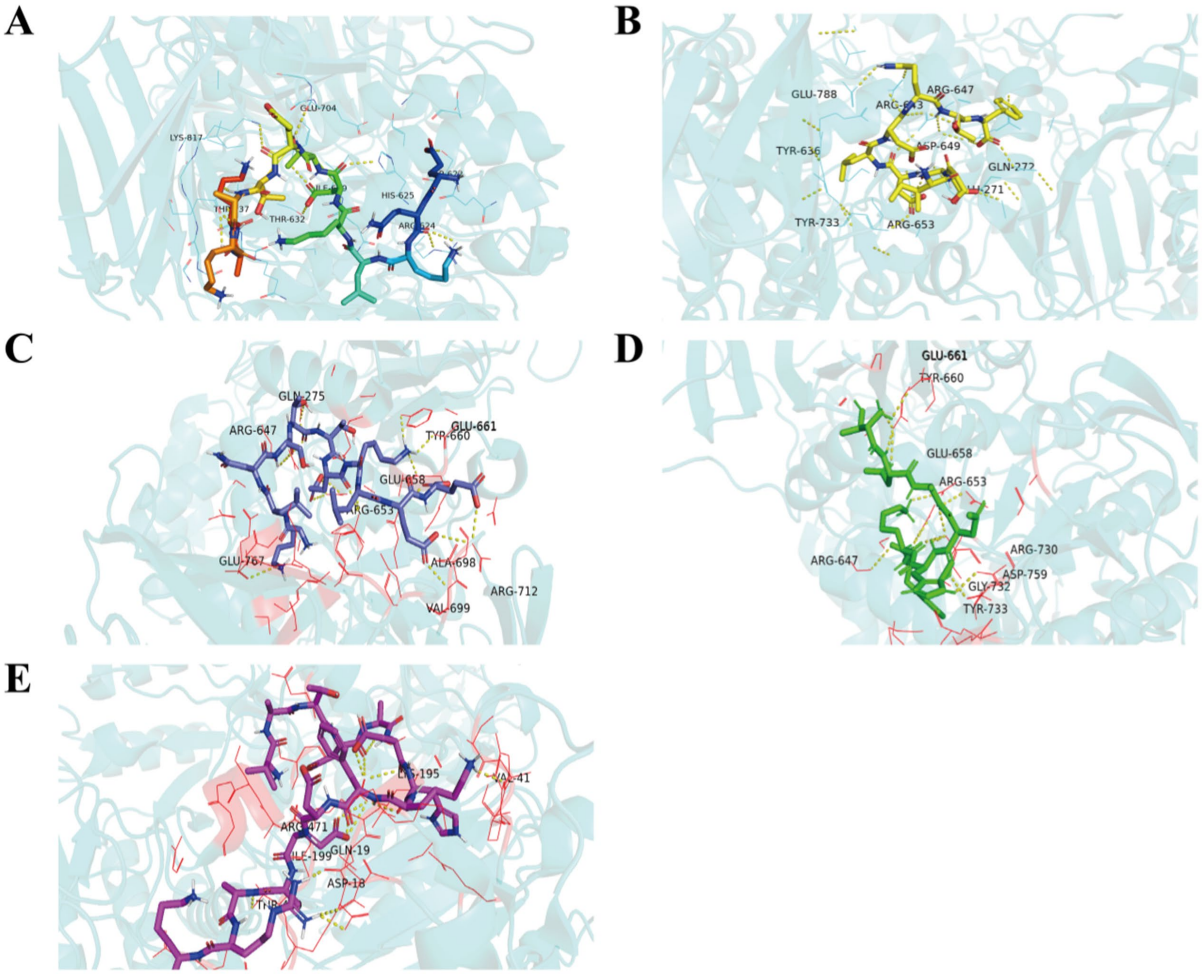
Hydrophobic interaction between EQKLKDTETAKAK **(A)**, KLNSTTEKLEE **(B)**, VATDADHKFDEAARK **(C)**, TDADHKF **(D)**, ISDDLDQTF **(E)** and amino acid residues of α-glucosidase.

Molecular docking, used to position computer-generated 3D structures of small ligands into receptor structures in various orientations, conformations and positions, has become an integral part of computational drug design and discovery (37, 38). Molecular docking is widely used in peptide activity by modeling the way molecules interact and predicting their binding modes and affinities. The lower the intermolecular binding energy, the higher the affinity of the peptide for the protein, and theoretically the higher the inhibitory activity of the peptide (39). The five peptides screened had docking binding energy scores ranging from −5.9 to −7.9 kcal/mol (Table 4), of which TDADHKF and KLNSTTEKLEE had the highest predicted binding energies of −7.9 kcal/mol and −7.2 kcal/mol, respectively. Figure 3 shows the hydrophobic interaction between peptides and amino acid residues of *α*-glucosidase. These results intuitively indicate that hydrogen bonding is the main driving force of the interaction, which is more consistent with previous studies (40, 41).

### 3.5 Activity validation of the synthetic peptides

**Figure 4:**
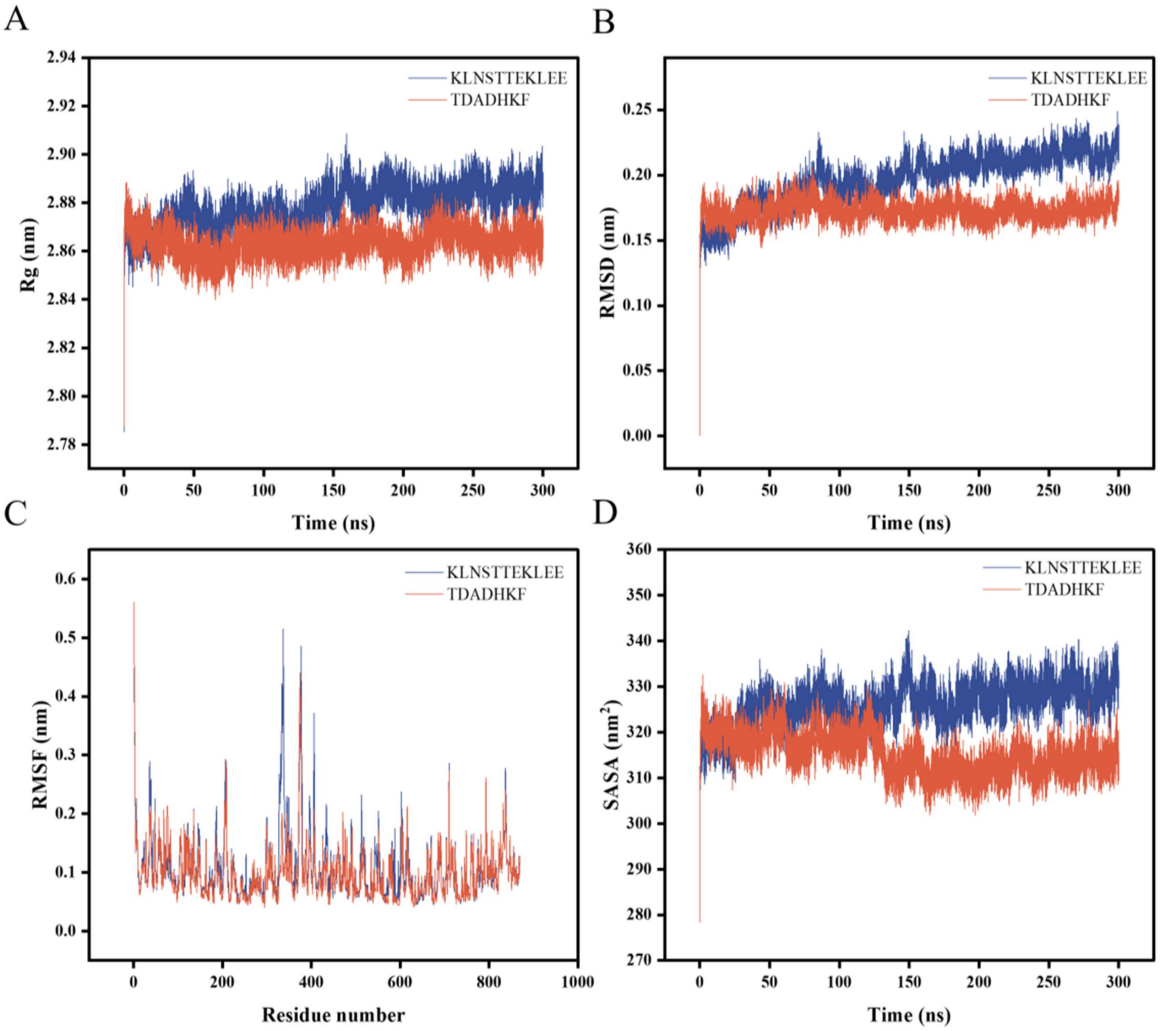
Molecular dynamics results of α-glucosidase with KLNSTTEKLEE and TDADHKF. **(A)** Rg; **(B)** RMSD; **(C)** RMSF; **(D)** SASA.

The stability and binding affinity between α-glucosidase and the two small molecules were further investigated using the GROMACS program. Stable complex systems were obtained through 300 ns molecular dynamics (MD) simulations, and the kinetic properties of the two complexes were derived by analyzing the trajectory data from these simulations. In the molecular dynamics study, each system was evaluated using several metrics, including the radius of gyration (Rg), root mean square deviation (RMSD), root mean square fluctuation (RMSF), and solvent accessible surface area (SASA). The results are presented in Figure 4. Gyrate is an indicator of the overall compactness of a protein and describes the distribution of system atoms along a specific axis (42). The results indicate that the Gyrate values of *α*-glucosidase with TDADHKF were stabilized and lower than those of KLNSTTEKLEE after 100 ns of binding to the two small molecules. This suggests that the binding of the small molecules resulted in a more compact protein structure. Root mean square deviation (RMSD) is an index used to assess structural changes in proteins (43). The results indicate that after 100 ns, the RMSD amplitude between the protein and TDADHKF was smaller compared to that of KLNSTTEKLEE. Furthermore, the RMSD value remained within a narrow range, suggesting that the binding of the protein to small molecules was relatively stable. Root mean square fluctuation (RMSF) is a metric used to assess protein dynamics (44). The results indicate increased residue flexibility in the critical region, with larger RMSF values observed for the bound portion in the 300–400 region. The RMSF of *α*-glucosidase with TDADHKF reaches a maximum value of 0.5 nm, while the unbound portion exhibits smaller RMSF values. These minor fluctuations suggest that the atoms in the unbound portion form a stable complex with α-glucosidase due to the strong intermolecular interactions that restrict their movement in molecular dynamics simulations. SASA is an index utilized to evaluate the surface area of proteins (45). The results indicate that the amplitude of the α-glucosidase and TDADHKF curves fluctuated slightly between 0 and 100 ns, after which there was a general decrease in amplitude. This trend suggests that the binding of small molecules leads to a reduction in the surface area of the proteins, implying that the interaction with small molecules renders the protein structures more compact. This finding is consistent with the results of the radius of gyration (Rg) analysis.

Analyzing the Rg, RMSD, RMSF, and SASA results of the two peptide-enzyme complexes mentioned above, we can conclude that TDADHKF is more effective than KLNSTTEKLEE in interacting with *α*-glucosidase. Furthermore, the interactions between the protein and small molecules exhibit stability, and the binding of these small molecules leads to a more compact protein structure.

### 3.6 Activity validation of the synthetic peptides

**Figure 5:**
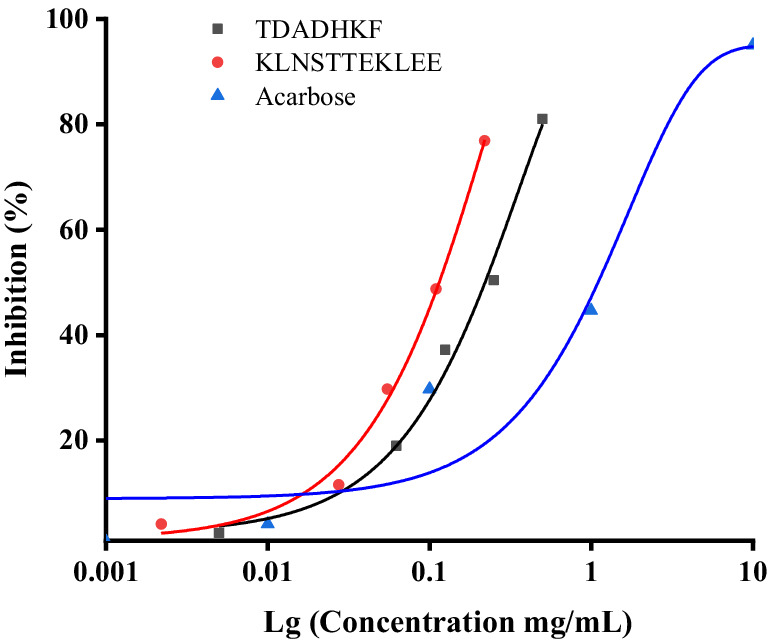
Inhibition activity of acarbose, KLNSTTEKLEE and TDADHKF.

After computer prediction, the two peptides with the highest docking score (KLNSTTEKLEE and TDADHKF) were subjected to further synthesis and activity validation, with the purity of the synthesized peptides exceeding 98%. Solubility tests under physiological conditions (pH 7.4, 37°C) revealed that both KLNSTTEKLEE and TDADHKF exhibited good solubility, consistent with the water-solubility predictions provided in Table 4. These results validate the accuracy of our initial screening process and highlight the potential of these peptides as therapeutic agents. The results show that both peptides had strong inhibitory effects on *α*-glucosidase with IC_50_ values of 144.89 μM and 136.96 μM for *α*-glucosidase, respectively, which showed higher effect than that of the positive control acarbose (with an IC_50_ value of 709.41 μM) (Figure 5). To further contextualize our findings, we compared the peptides identified in this study with previously reported α-glucosidase inhibitory peptides derived from both terrestrial and marine sources. Peptide WH, released from almond oil production residues, was stabilized in simulated gastrointestinal digestion and was able to maintain the IC_50_ value for α-glucosidase inhibition (17.03 ± 0.05 μmol/L) (46). Several studies have identified bioactive peptides from various marine sources with significant *α*-glucosidase inhibitory activities. For instance, Hu et al. (16) reported a peptide (LRSELAAWSR) from *Spirulina platensis* with an IC50 value of 134.2 μM, demonstrating its potential as an antidiabetic agent. Similarly, peptides derived from the Pacific oyster (*Crassostrea gigas*) (15) have shown promising inhibitory effects on α-glucosidase. More recently, Zhang et al. (47) identified peptides (LLDLGVP, AALEQTER, ILYGDFK, KAVGEPPLF, and GPAGPQGPR) from silver carp muscle hydrolysate with IC50 values ranging from 647.02 μM to 2665.46 μM.

These results demonstrate that the peptides from *Chlamys nobilis* adductor muscle have strong *α*-glucosidase inhibitory activities, comparable to or better than some marine-derived peptides and moderately lower than highly active terrestrial peptides. This comparison highlights the potential of *Chlamys nobilis* as a novel source of bioactive peptides with antidiabetic potential and underscores the importance of exploring marine proteins for the development of natural antidiabetic agents.

This study confirms that EHCA exhibits robust antioxidant and blood glucose-regulating activities in mice, and identifies multiple novel α-glucosidase inhibitory peptides from EHCA as potential adjuvants for hypoglycemic effects. This provides a new natural drug option for treating hyperglycemia and suggests that these peptides have potential applications for regulation of blood sugar levels. However, there are some limitations in this study, such as the lack of long-term effects assessment and the detailed investigation of peptide stability and bioavailability *in vivo*. Future studies should focus on exploring the long-term effects of EHCA in diabetic animal models or investigating the stability and bioavailability of the identified peptides in vivo. Furthermore, in vivo experiments and clinical trials remain warranted to validate the safety and efficacy of these peptides.

## 4 Conclusion

In the present study, we found that EHCA has strong *α*-glucosidase inhibitory activity (approximately 35%) and, at the same time, good antioxidant activity, as evidenced by DPPH radical scavenging of 55.45–80.02%, accompanied by concentration dependence. The results of animal tests show that EHCA had no significant effect on insulin secretion of mice; a certain concentration of EHCA could enhance the glucose tolerance of mice, significantly reduce MDA content, and improve SOD activity (*p* < 0.05). Highly inhibitory active peptides KLNSTTEKLEE and TDADHKF were obtained by LC–MS/MS and virtual screening techniques for EHCA with IC_50_ values of 144.89 μM and 136.96 μM, respectively. In summary, this study demonstrates the potential of EHCA as a natural source of bioactive peptides with strong α-glucosidase inhibitory and antioxidant activities. The identified peptides KLNSTTEKLEE and TDADHKF show promising inhibitory effects, highlighting their potential as novel antidiabetic agents. These findings provide a foundation for further exploration of EHCA and its peptides for the development of functional foods and therapeutic interventions.

## Data Availability

The original contributions presented in the study are included in the article/Supplementary material, further inquiries can be directed to the corresponding authors.
