# Supplementary figures and images for: Computational screening for novel α-glucosidase inhibitory peptides from Chlamys nobilis adductor muscle as a potential antidiabetic agent
Source: Front Nutr. 2025 Mar 24;12:1566107. doi: 10.3389/fnut.2025.1566107 (PMC11973072; doi:10.3389/fnut.2025.1566107)

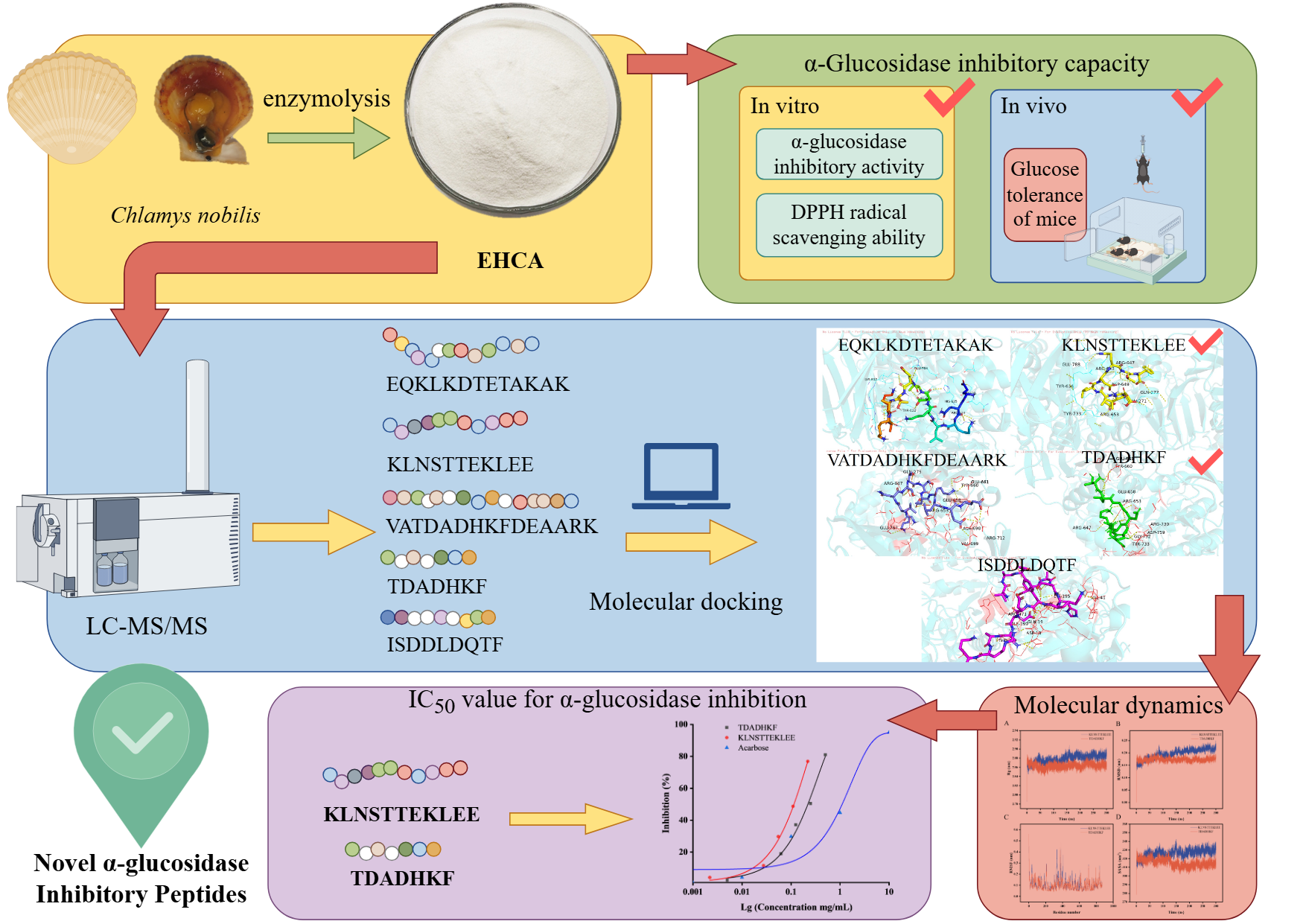

Supplement: Supplementary file 1 [file Image_1.PNG]
